# Hospital Needs

**Published:** 1912-09-28

**Authors:** 


### Hospital Needs.

### A NEW QUININE.

Messrs. Widenmann, Broicher and Co., London agents for Zimmer and Co., of Frankfurt, have submitted to us samples of a new quinine derivative, to which the name of hydroquinine hydrochloride has been given. This substance is prepared from true quinine, than which it contains two more hydrogen atoms per molecule. It does occur in very minute quantities in cinchona bark; but until the method of synthesising it from quinine itself was discovered, the difficulty of obtaining it prohibited its use. One of its most marked properties is the affinity of hydroquinine hydrochloride for water. Whereas quinine hydrochloride is soluble only to the extent of some 3 per cent. in water, hydroquinine is soluble in half its weight of water. This gives it a great advantage for hypodermic introduction. In toxicity there is little to choose; what difference there is, is in favour of the new product. It is not yet certain whether hydroquinine is superior to quinine in the treatment of malaria; but against trypanosomes the new remedy is stated to have a greatly augmented effect.

